# Divergent Role of Sphingosine 1-Phosphate in Liver Health and Disease

**DOI:** 10.3390/ijms19030722

**Published:** 2018-03-03

**Authors:** Burkhard Kleuser

**Affiliations:** Department of Toxicology, Institute of Nutritional Science, Faculty of Mathematics and Natural Science, University of Potsdam, Arthur-Scheunert Allee 114-116, 14558 Nuthetal, Germany; kleuser@uni-potsdam.de; Tel.: +49-33200-885301; Fax: +49-33200-885541

**Keywords:** sphingolipids, sphingosine kinase, fibrosis, non-alcoholic fatty liver disease, insulin resistance, liver fibrosis

## Abstract

Two decades ago, sphingosine 1-phosphate (S1P) was discovered as a novel bioactive molecule that regulates a variety of cellular functions. The plethora of S1P-mediated effects is due to the fact that the sphingolipid not only modulates intracellular functions but also acts as a ligand of G protein-coupled receptors after secretion into the extracellular environment. In the plasma, S1P is found in high concentrations, modulating immune cell trafficking and vascular endothelial integrity. The liver is engaged in modulating the plasma S1P content, as it produces apolipoprotein M, which is a chaperone for the S1P transport. Moreover, the liver plays a substantial role in glucose and lipid homeostasis. A dysfunction of glucose and lipid metabolism is connected with the development of liver diseases such as hepatic insulin resistance, non-alcoholic fatty liver disease, or liver fibrosis. Recent studies indicate that S1P is involved in liver pathophysiology and contributes to the development of liver diseases. In this review, the current state of knowledge about S1P and its signaling in the liver is summarized with a specific focus on the dysregulation of S1P signaling in obesity-mediated liver diseases. Thus, the modulation of S1P signaling can be considered as a potential therapeutic target for the treatment of hepatic diseases.

## 1. Introduction

**Figure 1:**
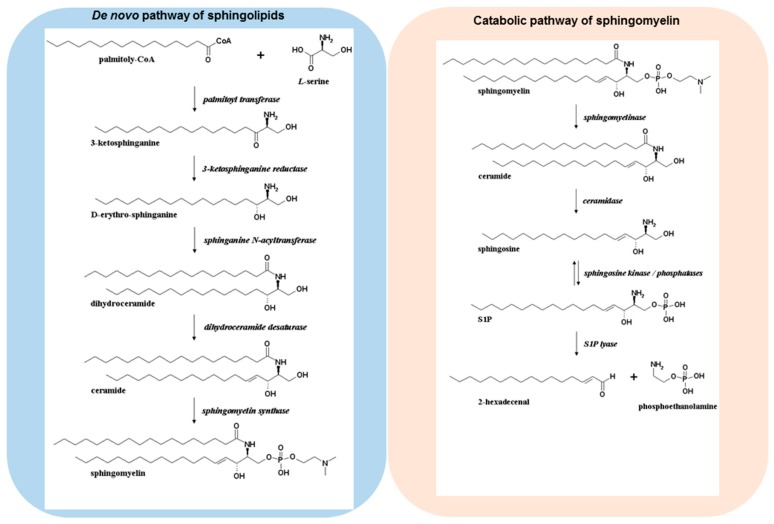
De novo pathway and degradation of sphingomyelin. Ceramides are formed through de novo biosynthesis or degradation of the cell membrane constituent sphingomyelin. Sphingosine kinases (SphKs) catalyze the formation of S1P from sphingosine. Irreversible cleavage of S1P occurs via S1P lyase leading to hexadecenal and phosphoethanolamine.

Over 20 years ago, sphingosine 1-phosphate (S1P) was discovered as a novel bioactive molecule, and it is now well established that S1P regulates a multitude of functions such as cell growth, survival, differentiation, migration, lymphocyte circulation, and immune cell regulation [1,2,3,4]. S1P can be formed via several pathways (Figure 1). De novo sphingolipid biosynthesis starts in the endoplasmic reticulum, where a set of enzymes leads to the formation of ceramides [5]. The sequential action of serine palmitoyltransferase, 3-ketodihydrosphingosine reductase, dihydro-ceramide synthase, and desaturase convert cytosolic serine and palmitoyl CoA molecules into ceramides, which are then incorporated into several complex sphingolipid derivatives via modulation at the primary hydroxyl group to form sphingomyelin, glycosphingolipids, or ceramide 1-phosphate [6]. In sphingolipid catabolic pathways, these complex molecules can be hydrolysed, which results in the formation of ceramides. The deacylation of ceramide species is achieved via ceramidases leading to the formation of sphingosine. The conversion of sphingosine to S1P occurs via two sphingosine kinases (SphK1 and -2). SphK1 is able to translocate from the cytosol to the plasma membrane in response to several stimuli such as growth factors, hormones, and cytokines [7]. The translocation of SphK1 facilitates the formation of S1P at the cell membrane and the subsequent transport to the extracellular environment [8,9]. Indeed, protein spinster homologue 2 (SPNS2) [10,11], as well as the ATP-binding cassette (ABC) transporters such as ABCA1, ABCC1, ABCG2 have been identified to transport S1P across the plasma membrane [12,13], where the bioactive molecule can bind and signal through a family of five G protein-coupled receptors (S1PR1–5) in an autocrine or paracrine manner [14]. S1PR1–3 are ubiquitously expressed throughout the body, whereas S1PR4 is predominantly expressed in the immune system, and S1PR5 in the central nervous system and the spleen. Because of the specific receptor expression profile, S1P is able to orchestrate complex physiological responses. 

In contrast to SphK1, SphK2 is mainly localized in the nucleus and in the inner mitochondrial membrane increasing the S1P concentration within the nucleus, where the lipid mediator can act intracellularly [15]. The intracellular targets of S1P include histone deacetylases (HDACs) and tumour necrosis factor (TNF) receptor-associated factor 2 [16,17]. SphK2 directly interacts with histone H3 and HDACs, followed by the formation of S1P. The formed S1P binds to and inhibits both HDAC1 and HDAC2, suggesting that the nucleus-generated S1P via SphK2 influences the dynamic balance of histone acetylation and thus the epigenetic modulation of specific target genes.

The terminal degradation of sphingolipids is initiated by an irreversible cleavage of S1P via S1P lyase, leading to the formation of hexadecenal and phosphoethanolamine. The aldehyde can be further metabolized to palmitoyl CoA, however it has been indicated that hexadecenal is an active compound that can form adducts with cellular nucleophiles such as proteins and glutathione [18,19]. 

The liver is one of the vital organs of the body with a variety of multiple functions. On the one hand, the liver is responsible for the synthesis of several proteins and for the detoxification of various metabolites and xenobiotics. On the other hand, it exhibits a distinguished role in the metabolism of carbohydrates, proteins, amino acids, and lipids. This review presents a brief background about the liver as a crucial organ for modulating S1P levels in the plasma, which is important for endothelial barrier function and immune cell trafficking. Further, it is illustrated that S1P and enzymes involved in S1P metabolism possess a pivotal role in liver physiology and pathophysiology. Many different disease processes occur in the liver, including infections, cancers, fibrosis, and metabolic dysfunctions. The divergent role of S1P in liver physiology and disease is highlighted.

## 2. Regulation of Plasma S1P Content via Liver-Formed Apolipoprotein M

The egress of lymphocytes from the lymphoid tissues to the blood occurs in response to a S1P gradient (high in blood and low in lymphoid tissue) via the receptor subtype S1PR1 [20,21]. Albumin and apolipoprotein M (apoM) are the main carriers of S1P in the circulation [22]. Plasma apoM mainly associates with high-density lipoproteins (HDL) (more than 90%) and to a minor extent with low-density lipoproteins, very low-density lipoproteins, and chylomicrons [23]. HDL-associated apoM carries about 65% of plasma S1P, whereas albumin is able to transport approximately 30% of S1P [24]. As hepatocytes express and secrete the majority of circulating apoM, the liver plays a crucial role in maintaining the S1P gradient between blood and lymphoid tissues. Hepatic apoM serves as a chaperone to transport S1P through the vascular system and therefore has a great impact on S1P levels in the blood [25]. In contrast to albumin, apoM binds S1P with a high affinity in a hydrophobic binding pocket of the lipocalin domain, indicating that apoM is the preferred transporter of the bioactive lipid [26]. ApoM released by hepatocytes correlates with S1P concentrations in the plasma [27]. Accordingly, apoM-deficient mice display a diminished plasma S1P level of about 50%. The reduced plasma content of S1P is the result of no detectable S1P in HDL and not of modified S1P levels in the albumin fraction [22]. Conversely, hepatic overexpression of apoM using an adenoviral vector strategy in mice indicates an increased S1P level in plasma [28]. Red blood cells, platelets, endothelial cells, and hepatocytes are important cell types to maintain S1P contents in plasma and lymphoid tissues [29]. Red blood cells constitute approximately 95% of total blood cells and are therefore the main sources of blood S1P [30]. However, the exact mechanism that triggers S1P release from erythrocytes to apoM-associated HDL and albumin is not well investigated. Nevertheless, it has been discovered that erythrocytes export S1P more efficiently to HDL than to albumin, especially when apoM is present in HDL [30]. Treatment with MK-571, an inhibitor of ABC transporters with a preference for inhibition of ABCC1, is able to reduce the export of S1P from human erythrocytes to apoM, indicating the crucial role of ABC transporters for the interaction of red blood cells with apoM [30].

Besides erythrocytes, endothelial cells are essential to preserve a constant S1P gradient [31]. It has been shown that ABC transporters and SPNS2 are able to export S1P from endothelial cells into the blood and the lymph [32]. Abrogation of SPNS2 in mice indicated a reduced S1P content in the blood of approximately 20% and in the lymph of more than 80%, demonstrating the crucial role of this cell type in maintaining S1P levels especially in the lymph [33]. Furthermore, it is of functional importance whether S1P is associated to apoM or albumin [34]. ApoM-associated S1P induces an activation of S1PR1 followed by internalization and recycling of the receptor to the plasma membrane. In contrast, albumin-associated S1P leads to internalization and subsequent degradation of the S1PR1 receptor subtype [34]. The divergent interaction of apoM-S1P and albumin-S1P with the S1PR1 receptor subtype is important to modulate the maintenance of the endothelial barrier function. Several studies indicate that S1P enhances the barrier function of the vascular endothelium via S1PR1 [20,34]. ApoM-deficient mice show an increased endothelial permeability in the lung, although S1P levels bound to albumin are unchanged [22]. These data suggest that apoM-associated S1P is the distinguished mediator that increases endothelial barrier integrity, whereas albumin serves as a reservoir protein for the transportation of S1P [34]. A tight interaction between apoM and the S1PR1 enables S1P to activate and signal in an optimal manner through this receptor subtype [34]. These data implicate that plasma levels are necessary to preserve essential physiological functions of S1P, such as immune cell trafficking and modulation of cell integrity, and hepatocytes are important regulators of S1P contents in the blood because of the formation of apoM.

## 3. S1P and Hepatic Insulin Resistance

Insulin resistance is a multifaceted metabolic condition characterized by the fact that insulin-sensitive tissues are not able to respond adequately to physiological concentrations of insulin [35]. There is a strong association between insulin resistance and the appearance of type 2 diabetes, both reaching epidemic proportions [35]. In the liver, insulin resistance is accompanied by an increased hepatic glucose formation due to an attenuated efficacy of insulin to inhibit hepatic gluconeogenesis and glycogenolysis and to stimulate glycogen synthesis [36]. 

Both SphK1 and SphK2 play a fundamental role in hepatic insulin resistance. It has been shown that high-fat diet (HFD)-fed SphK1-deficient mice develop an evident diabetes, whereas in wildtype mice only a glucose intolerance and a compensatory hyperinsulinemia are detected [37]. However, β-cell degeneration is responsible for this effect, indicating a protective role of S1P on β-cell survival [37]. In congruence, the specific adenoviral delivery of SphK1 to the liver is able to diminish blood glucose levels and dyslipidemia in the KK/Ay mouse model of type 2 diabetes. Moreover, SphK1 gene delivery significantly enhances the phosphorylation of insulin signaling kinases such as Akt and glycogen synthase kinase (GSK) 3β in the livers of the diabetic animals [38]. Conversely, another mice study indicated that overexpression of SphK1 in the liver does not affect glucose tolerance, independently of whether the animals are fed with a low-fat diet or a high-fat high-sucrose diet [39]. 

Recently, it has been demonstrated that SphK2 is highly expressed in the liver. In primary hepatocytes, adenoviral SphK2 expression elevates intracellular S1P levels and stimulates Akt phosphorylation. Hepatic overexpression of SphK2 in the KK/Ay mouse model leads to elevated S1P levels and ameliorates glucose intolerance and insulin resistance in response to a HFD [40]. 

**Figure 2:**
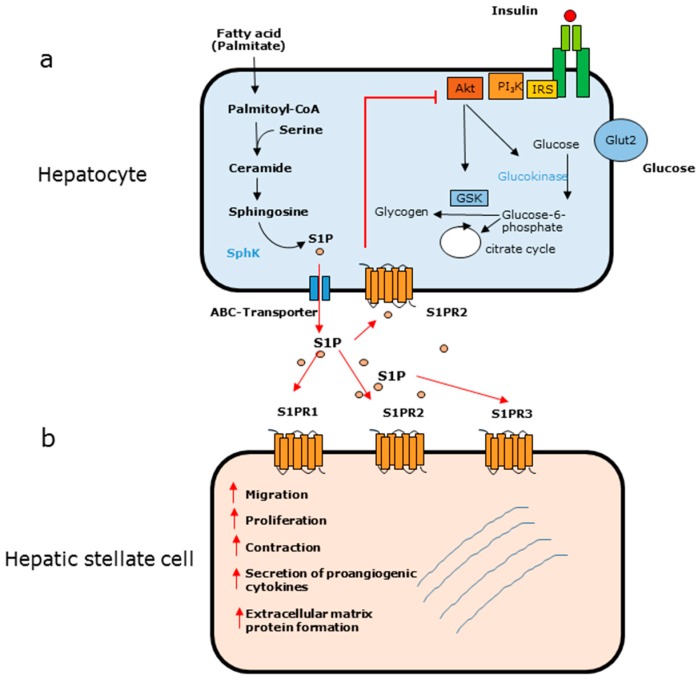
(Adapted from [45]) (**a**) Effect of palmitate in hepatocytes. Palmitate is metabolized to S1P, which is released into the extracellular environment. S1P binds to the S1PR2, which results in an inhibition of Akt and a diminished action of insulin signaling leading to insulin resistance. (**b**) Effect of S1P on hepatic stellate cells. In response to S1P via different S1PR subtypes, quiescent HSCs are activated and undergo a phenotypic change to myofibroblast-like cells.

An apparent contrary role of S1P in hepatic insulin signaling has been described. The treatment of obese mice with myriocin, an inhibitor of serine palmitoyl transferase, is accompanied by an improvement of glucose tolerance. Myriocin attenuates the increase of ceramide formation, suggesting a fundamental role of ceramides in insulin resistance [41]. However, it has been shown that fumonisin B1, an inhibitor of ceramide synthases, diminishes ceramide levels in HepG2 cells, which does not improve insulin resistance [42]. In this context, it must be mentioned that in response to fumonisin B1, the levels of S1P are increased in hepatocytes [42]. Elevated S1P levels are a key feature not only in diabetic animal models but also in patients suffering from type 2 diabetes, suggesting that extracellular S1P modulates insulin signaling [43,44]. A recent study indicated that S1P counteracts the insulin pathway and contributes to hepatic insulin resistance in vitro as well as in vivo (Figure 2) [45]. This study revealed that the fatty acid palmitate is metabolized to S1P in primary rat and human hepatocytes. Additionally, S1P levels are increased in the liver of HFD-fed New Zealand obese mice [45]. The inhibition of insulin signaling by S1P seems to be a receptor-dependent process. The S1PR2, which is highly expressed in hepatocytes, is responsible for the inhibition of insulin signaling, as pharmacological abrogation of this receptor almost completely abolishes the ability of S1P to diminish insulin action in vitro and in vivo [45]. This is consistent with the fact that the immunomodulator FTY720-phosphate, which acts on all S1P receptors except for S1PR2, does not diminish insulin signaling [45]. 

Conversely, S1P has been discussed as a protective molecule in the action of adiponectin, whose relevance to insulin sensitivity has been well elucidated in the last years [46]. As a promoter of ceramide degradation, adiponectin alters bioactive sphingolipid species [47]. On the one hand, adiponectin diminishes the accumulation of ceramide, whereas on the other hand adiponectin stimulates the formation of S1P. This change of the ceramide/S1P rheostat has been discussed to improve the action of insulin [47]. Such a modulation of the ceramide/S1P balance occurs also in acid sphingomyelinase-deficient mice. These mice show an improved glucose tolerance and an increased formation of glycogen as well as an enhanced lipid accumulation in the liver [48]. 

## 4. S1P and Non-Alcoholic Fatty Liver Disease

Non-alcoholic fatty liver disease (NAFLD) is the most common hepatic disorder in Western countries that is not associated with hepatic virus infection or chronic alcohol consumption [49]. NAFLD is characterized by hepatocellular lipid accumulation, steatosis, and an increase of liver enzymes in the plasma [50]. While NAFLD is a reversible disease, 10–25% of NAFLD patients develop a more severe form of NAFLD, namely, nonalcoholic steatohepatitis (NASH) [49]. NASH is one of the leading causes of cirrhosis, and up to 25% of adults with NASH may have cirrhosis [49,51]. 

There is a close association between hepatic steatosis and the development of insulin resistance. This relationship can be explained by hyperinsulinemia, which occurs as one of the first signs of insulin resistance, and this is connected with a transcriptional upregulation of genes involved in lipogenesis [52]. Several studies have indicated that fatty acid uptake and fat accumulation in the liver leads to the formation of bioactive lipid intermediates that are able to interrupt insulin signaling, hereby accelerating insulin resistance [53,54]. The generation of diacylglycerols has been discussed as a crucial mechanism in the interruption of insulin signaling [53]. However, saturated and unsaturated fatty acids differ in their ability to counteract the insulin pathway [54]. This can be explained by the circumstance that the saturated fatty acid palmitate is the precursor molecule for the de novo synthesis of ceramides. Additionally, saturated fatty acids are agonists for toll-like receptor (TLR)4, and the stimulation of this receptor is associated with the upregulation of genes driving ceramide biosynthesis [55]. Numerous studies have described a crucial role for ceramides in the development of insulin resistance [56,57,58]. On the molecular level, it has been shown that ceramides activate atypical protein kinases C (PKC) such as PKCζ and attenuate Akt signaling, and these pathways cause the interruption of insulin signaling [59]. A mouse model of NAFLD, characterized by an increased hepatic lipid accumulation, has indicated that treatment with the de novo sphingolipid inhibitor myriocin results in a reduced hepatic steatosis [60]. Myriocin-treated animals on a HFD show not only a decreased lipid accumulation but also a diminished immune cell infiltration [56]. These data suggest that ceramides or further sphingolipid derivatives are involved in lipid accumulation and progression of NAFLD. Indeed, more recently, it has been demonstrated that SphK and the subsequent formation of S1P play a pivotal role in the modulation of lipid accumulation and lipid storage [61]. A lipidomic readout of disease progression in a diet-induced mouse model of NAFLD has indicated that sphingosine, S1P, dihydrosphingosine, and dihydrophingosine 1-phosphate increase by week 16 after a high-fat high-cholesterol diet [61]. It has been observed that lipid oversupply induces the activation of SphK1 in vitro as well as in vivo, which is connected with an increased lipid storage [62,63]. Overexpression of SphK1 or treatment with S1P significantly enhances hepatic lipid storage, which is inhibited when S1P receptors S1PR2 and S1PR3 are downregulated via siRNA [62]. In conclusion, SphK1-deficient mice show a reduced manifestation of hepatosteatosis under diet-induced obese conditions compared to wild type mice. It has been suggested that S1P-induced lipid accumulation is mediated via upregulation of peroxisome proliferator-activated receptor γ (PPARγ) and its target genes, as the downregulation of PPARγ is able to abolish the prosteatotic effect of the bioactive lipid mediator S1P [62]. However, S1P seems to be involved in the progression of NAFLD to NASH [64]. A mouse model of NAFLD has revealed that the activation of NFκB elevates cytokine production and that immune cell infiltration occurs in response to a high saturated fat feeding. Most interestingly, SphK1-null mice are protected from these pro-inflammatory consequences [64]. It has been demonstrated that the S1PR1 is responsible for the progression of NAFLD to NASH, as saturated fat-induced NFκB signaling and elevation of TNF-α in HepG2 cells is abrogated by targeted knockdown of this receptor subtype [64]. Consistent with these results, an elevation of SphK1 can be observed in livers from humans with NAFLD, indicating the clinical significance of the SphK1–S1P axis in the development and progression of the disease [64]. At the moment, there are no approved pharmacologic therapies for NASH. In an attempt to treat NASH, a mouse study has been performed using FTY720, which can be phosphorylated to FTY720-phosphate acting as a functional antagonist on the S1PR1 receptor subtype [65]. FTY720-treated mice with NASH show a decreased lipid accumulation and a reduction of immune cell infiltration indicating a preclinical rationale for studying this drug in human NASH [66]. While SphK1 contributes to the development of NAFLD and NASH, a controversial role has been described for SphK2. Hepatic overexpression of SphK2 in mice on a HFD leads to an upregulation of fatty acid oxidizing genes and increases fatty acid oxidation in the liver. Consequently, the hepatic accumulation of lipid droplets is reduced in HFD-fed mice overexpressing SphK2 [40]. In congruence, an opposite action occurs when SphK2 is downregulated. SphK2 (−/−) mice rapidly develop fatty livers on a HFD, indicating the importance of SphK2 in regulating hepatic lipid metabolism [67]. It has been suggested that SphK2 activation is able to increase nuclear S1P formation, which inhibits specific HDACs causing an elevated acetylation of histones and thereby an upregulation of genes encoding nuclear receptors and enzymes involved in lipid metabolism [67].

## 5. S1P and Liver Fibrosis

Liver fibrosis is a result of chronic liver injury and a serious problem, as it can lead to cirrhosis and liver failure [68]. An excessive deposition of collagen and other matrix proteins into the extracellular environment is one of the key features of this disease. Hepatic stellate cells (HSCs), which reside in the space of disse, are known to act as a central player in the initiation and progression of liver fibrosis, as they undergo a phenotypic change from quiescent to myofibroblast-like cells in the initial phase of liver fibrosis [69]. Additionally, bone marrow-derived cells are able to migrate to injured liver tissue, where they transdifferentiate into myofibroblasts and contribute to liver fibrosis [70]. Reactive oxygen species and apoptotic cells are involved in HSC activation resulting in the formation of extracellular matrix proteins. Nevertheless, it is now well accepted that S1P plays a pivotal role in the activation of HSCs (Figure 2). Thus, it has been shown that S1P is able not only to stimulate HSC proliferation and migration in vitro, but also to induce the upregulation of extracellular matrix proteins such as alpha-smooth muscle actin (α-SMA) and collagen I and III [71,72,73]. Additionally, S1P participates in HSC-mediated angiogenesis, a critical process in liver fibrosis. On the one hand, HSCs secrete proangiogenic cytokines such as angiopoietin 1 and vascular endothelial growth factor in response to S1P [74]. On the other hand, the angiogenic platelet-derived growth factor is able to stimulate SphK1 in HSCs [75]. Furthermore, S1P affects the contractility of HSCs, which may result in an increased portal vein pressure [76]. This is of interest, as portal hypertension is a severe complication of liver fibrosis.

Although S1P seems to be a crucial molecule in the development of liver fibrosis, studies are not consistent whether S1P concentrations are increased in the tissues of patients with liver fibrosis. Fibrotic liver samples, obtained from liver resections due to hepatocellular carcinoma (HCC), exhibit no enhanced S1P levels [77]. Nevertheless, this study has shown that the S1P transporter SPNS2 is upregulated in such tissues, suggesting an augmented release of S1P into the extracellular environment. Another study, in which human fibrotic samples were obtained from the livers of patients undergoing liver transplantation, has indicated that S1P tissue levels are increased [78]. Similarly, an enhancement of S1P concentrations in the liver has been detected in animal models of liver fibrosis [74,79]. In vivo studies have indicated that S1P is synthesized in hepatocytes in response to palmitate and released into the extracellular environment leading to the activation of HSCs [73]. This is of interest, as ectopic lipid accumulation in hepatocytes is a risk factor for the progression of liver fibrosis. Furthermore, it has been indicated that increased levels of S1P are detectable in the serum of patients suffering from hepatitis C-induced liver fibrosis [80].

Both receptor-independent and receptor-mediated actions contribute to the profibrotic action of S1P. A receptor-independent action has been described as intracellular S1P possesses a critical function in the transforming growth factor (TGF)-β-induced expression of collagen, which is required for human fibrosis development [81]. However, most studies postulate a receptor-mediated action of S1P to promote liver fibrosis. A common experimental model of liver fibrosis is bile duct ligation, which causes periportal biliary fibrosis, cholestasis, and an increased proliferation of biliary epithelial cells, enhancing the formation of extracellular matrix proteins. Knockout of the S1PR2 in this model protects mice from the development of fibrosis, suggesting a crucial role of this receptor subtype in the development of liver fibrosis [77,82]. In analogy, the S1PR2 antagonist JTE-013 is able to diminish liver fibrosis. However, several studies have indicated that the S1PR3 is the critical receptor subtype involved in liver fibrosis. In fact, S1P-induced migration and activation of HSCs occur in response to the activation of the S1PR3 [73,78]. Further, S1PR3 exhibits a critical function regarding the recruitment of bone marrow-derived cells to the liver tissue. Silencing of the S1PR3 diminished not only the ability of bone marrow-derived cells to migrate to the liver but also their transdifferentiation into myofibroblast-like cells [83]. It has been demonstrated that the S1PR3 is upregulated during liver fibrosis [78]. Most recently, the mechanism of this upregulation has been elucidated [84]. HuR, an RNA-binding protein, is enhanced during liver fibrosis via S1P, as the bioactive lipid mediator induces phosphorylation and cytoplasmic translocation of HuR. This is accompanied by an increased expression of the S1PR3 resulting from a stabilization of its mRNA by the RNA-binding protein HuR [84]. 

Although most studies indicate a profibrotic action of S1P, it should be considered that basal levels of S1P, especially if bound to apoM, are beneficial in liver fibrosis. The liver has the ability to regenerate after damage, and the resection of 70% of liver tissue by partial hepatectomy is associated with a rapid regrowth of a functional liver mass [85]. Nevertheless, a coordinated interaction between hepatocytes, HSCs, vascular endothelial cells, and hematopoietic cells is required [86]. The disturbance of this coordinated interaction may result in a dysfunctional regeneration leading to the formation of fibrotic tissue. Indeed, in mice lacking HDL-S1P, liver regeneration after partial hepatectomy as a model to induce fibrosis is associated with a defective vascularity. The stimulation of the S1PR1 with pharmacological agonists is able to abolish the vascular dysfunction and alleviates the formation of fibrotic tissue, indicating the imperative need of S1P for a physiological regeneration of liver tissue [87]. Moreover, it has been indicated that partial hepatectomy is connected with the formation of exosomes from liver cells, which are membrane nanovesicles released into the extracellular environment upon fusion of multivesicular bodies with the plasma membrane [88]. Actually, exosomes produced by hepatocytes, but not by other liver cell types, stimulate hepatocyte proliferation in vitro and enhance liver regeneration in vivo. The molecular mechanism of the regenerative effect can be explained with the fact that such exosomes possess the synthetic machinery to produce S1P [89,90]. These data suggest that S1P, formed in exosomes, contributes to liver repair and regeneration after injury.

Taken together, S1P plays a divergent role in liver fibrosis, and further studies are important to define the exact role of the bioactive molecule and its receptors in the different stages of liver fibrosis.

## 6. S1P and Viral Infections

Hepatic dysfunction is a key feature of dengue virus (DENV) infection [91]. The incidence of DENV infection has been dramatically increased over the last decades, causing a fundamental disease burden in tropical and subtropical regions of the world [92]. The virus is spread by the bite of female mosquitoes of the species *Aedes aegypti* and *Aedes albopictus*. From the site of infection, the virus spreads to numerous organs via the blood and the lymphatic system [91]. Especially in severe cases of DENV infection, high levels of viremia can be detected in the liver. In patients with hepatomegaly, viral antigens are present in hepatocytes as well as in Kupfer cells [93,94]. An ensuing consequence of hepatocyte infection by DENV is cellular apoptosis, which has been demonstrated in in vivo as well as in vitro experiments [95,96]. Both the intrinsic and extrinsic apoptotic pathways are activated in response to DENV infection [97]. It has been indicated that SphK1 as well as SphK2 are modulated in response to DENV infection in an opposite manner. On the one hand, tumour necrosis factor alpha (TNF-α) stimulation of DENV-infected cells during productive infection leads to enhanced death by caspase-3-mediated apoptosis, which is accompanied by a reduced SphK1 activity [98,99]. On the other hand, the activation of SphK2 contributes to DENV-provoked apoptosis in hepatocytes [100]. Pharmacological inhibition of SphK2 or downregulation via siRNA reduced caspase-3 as well as caspase-9 activity in DENV-infected cells, indicating a pro-apoptotic role of SphK2 via the intrinsic pathway. 

Vascular leakage is another hallmark of severe DENV infection, which is associated with the development of shock and multiorgan failure [101]. As S1P is essential for endothelial barrier integrity, it has been examined whether S1P levels are decreased in patients with DENV infection. Indeed, two studies indicate that during the different stages of DENV infection, a significant decrease of plasma S1P levels occurs, which correlates with DENV-induced plasma leakage [102,103]. In addition, apoM levels are decreased in response to DENV infection [103]. Accordingly, it can be speculated that the reduced apoM concentration is at least in part responsible for the diminished S1P plasma concentration. Therefore, the modulation of the levels of S1P and of its receptors may be a novel therapeutic strategy to prevent plasma leakage after DENV infection.

According to the World Health Organization, approximately 500 million individuals worldwide are chronically infected with either hepatitis B virus (HBV) or hepatitis C virus (HCV). Almost 1 million people die each year, as chronic infections may cause liver cirrhosis and HCC [104]. It has been indicated that S1P plasma concentrations in patients with HCV are reduced compared to healthy subjects with the same hemoglobin concentration [105]. This is in accordance with studies showing that SphK1 is inhibited after infection with bovine viral diarrhea virus (BVDV), a close relative of HCV [106]. The nonstructural protein NS3 from BVDV, which is a cleavage product of the HCV polyprotein, binds to and inhibits the catalytic activity of SphK1. Overexpression of SphK1 significantly diminishes the induction of apoptosis in cells infected with cytopathogenic BVDV, which indicates that inhibition of SphK1 is a critical factor in viral cytopathogenesis [106]. In contrast to SphK1 that promotes viral replication, SphK2 possesses an opposing effect on viral replication [107]. It has been indicated that SphK2 promotes lipid peroxidation [107]. Multiple HCV genotypes are exquisitely sensitive to oxidative membrane damage, a property distinguishing them from other pathogenic RNA viruses [107]. Especially, HCV replicase is highly sensitive to endogenous lipid peroxidation. SphK2 upregulates lipid peroxidation with a subsequent inhibition of HCV replicase, thereby limiting virus replication [107]. 

It has been shown that, besides a reduction of S1P plasma levels, a significant increase of sphingosine and sphinganine occurs in patients with chronic HCV infection [80,108]. Interestingly, an association with the severity of liver fibrosis was observed for the lipid mediators sphingosine and sphinganine in the HCV patients, suggesting that these sphingolipids may be used as novel biomarkers for the progression of HCV infections. 

In contrast to observations in chronic HCV infection, HBV patients show an increased serum level of S1P [80]. Indeed, the multifunctional HBV X protein (HBx), which can be detected with a high frequency in liver cells from chronic HBV patients, induces an upregulation of SphK1 through the transcription factor AP2α, as there is an AP2α binding site in the promoter region of SphK1. This process is accompanied by an increased proliferation of human hepatoma cells [109]. While chronic infection with the HBV has been directly linked with the development of HCC, it has been speculated that activation of SphK1 may be involved in this process. 

## 7. S1P and Hepatocellular Carcinoma

HCC is a primary malignancy of the liver and arises mainly in patients with underlying chronic liver disease and cirrhosis [110,111]. HCC is the third leading cause of cancer deaths worldwide, with over 500,000 people affected. The geographic distribution of HCC mortality is comparable with that of its incidence; both are high in Asia and Africa [111]. Most HCC patients have severe liver dysfunction related to disorders such as chronic HBV or HCV viral infections, alcohol abuse, and metabolic diseases. Even though advances have been made to improve its diagnosis and management, the five-year survival of HCC is less than 50% [111,112].

There is accumulating evidence that S1P and enzymes involved in S1P metabolism are associated with the development and progression of HCC. A strong correlation between S1P levels in plasma and the growth of HCC has been detected [80,113]. The serum levels of S1P are increased in patients with HCC, compared with either patients with cirrhosis or healthy subjects [80].

A dysregulation of the enzymes modulating S1P metabolism has been detected in liver tissues from HCC patients. In a recent study, the expression of SphK1 was examined in 127 formalin-fixed, paraffin-embedded HCC tissues using immunohistochemistry, and its clinical implications and prognostic significance were examined [114]. This study has revealed that SphK1 is significantly higher in HCC tissue than in normal tissue. In addition, SphK1 expression is significantly associated with tumor size, tumor stag,e and histological differentiation. Patients showing a low SphK1 expression had higher overall survival and recurrence-free survival rates compared with those showing a high expression of SphK1 [114]. This result is in agreement with another study, in which SphK1 expression was measured in tissues of 77 patients with HCC who underwent surgical treatment. The study has indicated that SphK1 mRNA is increased in human HCC tissues compared with adjacent non-tumorous tissues [115]. Additionally, the expression of SphK2 is increased. The mRNA levels of both SphK1 and Sphk2 correlates with poorer differentiation in HCC and microvascular invasion in HCC tissues. Furthermore, the increased mRNA expression of SphK2 is a risk factor for intra- and extrahepatic recurrence [115]. 

These data suggest that SphK1 and SphK2 play a pivotal role in the pathophysiology of HCC. This is consistent with in vitro studies indicating that SphK1 promotes HCC proliferation, migration, and epithelial–mesenchymal transition, crucial steps involved in HCC tumor progression and metastasis [116,117,118]. The downregulation of SphK1 in HCC cell lines, including hepatoblastoma G2 and HCC-9724, has indicated that cell proliferation is decreased in the absence of SphK1, providing evidence that Sphk1 promotes HCC cell proliferation and is involved in tumor progression [117].

However, despite the increased mRNA expression of SphK1 and SphK2 in HCC tissues, S1P is even lower in human HCC tissues than in adjacent non-tumorous tissues [115]. Two different mechanisms may be responsible for this unexpected finding. On the one hand, an enhanced release of S1P from HCC tissue into the extracellular environment can occur [77]. On the other, an augmented degradation of S1P seems possible [115]. In this context, it has been demonstrated that the mRNA expression of S1P lyase is increased in HCC tissues compared with non-tumorous tissues [115]. The enhanced expression of S1P lyase in HCC tissues correlates with poorer HCC differentiation, suggesting that S1P lyase may play a pivotal role in the pathophysiology of HCC [115]. Furthermore, in vitro studies have shown that abrogation of S1P lyase expression via siRNA leads to diminished proliferation and invasion, whereas overexpression of S1P lyase causes enhanced proliferation of HCC cell lines [115]. This finding is of interest, as in colon cancer cells S1P lyase possesses an opposite role on tumor cell proliferation and survival [119]. However, a stimulatory role of S1P lyase on cell proliferation has been reported in mouse embryonic carcinoma cells with various expression levels of SphK1 and S1P lyase. The overexpression of both SphK1 and S1P lyase is connected with a stronger mitogenic effect compared with the overexpression of SphK1 only or to low levels of S1P lyase [120]. Additionally, it can be speculated that an increased expression of S1P lyase is accompanied by an enhanced formation of the cleavage product hexadecenal. As it has been indicated that hexadecenal is a reactive molecule that can form adducts with cellular nucleophiles such as liver proteins, further studies are of interest investigating whether the protein adduct formation of hexadecenal is connected with a dysfunction of hepatic liver proteins [19]. 

One of the key mechanisms driving the generation of HCC seems to be a dysregulation of the DNA damage response (DDR). The DDR is an entangled cellular network including multiple DNA repair pathways, damage tolerance processes, and cell-cycle checkpoints to safeguard genomic integrity. Several studies identified SphK1, SphK2, and S1P lyase as specific players in DDR. SphK1 protein levels are proteolysed in response to genotoxic stress in a p53-dependent manner, and this has been associated with the modulation of cell survival and inflammatory responses [121]. The p53 protein is a crucial tumor suppressor, and mutation in p53 is one of the main genetic alterations found in HCC [122]. Importantly, deletion of SphK1 in p53-null mice completely abrogates thymic lymphomas in these mice. The mechanism of p53 tumor suppression by loss of SphK1 is accompanied by increased expression of cell-cycle inhibitors and tumor cell senescence [121]. It has been reported that SphK2 is essential for the regulation of histone acetylation, leading to an epigenetic modification of key genes encoding enzymes and receptors that are involved in nutrient metabolism. A dysregulation of SphK2 activity may be related to the DDR and liver carcinogenesis. Besides SphK1 and SphK2, the S1P lyase functions as a modulator of the DDR [123]. S1P lyase modulates the kinetics of DNA repair as well as the speed of recovery from G2 cell-cycle arrest. Moreover, it has been demonstrated that the downregulation of S1P lyase leads to increased DNA repair kinetics in vitro [124]. The detailed mechanisms of how S1P and its metabolizing enzymes are involved in the modulation of HCC remain unclear. Further investigations are required to elucidate the specific role of S1P in HCC, which may create new therapeutic strategies for patients with HCC.

## 8. Conclusions

The liver is an important organ for regulating S1P levels in the blood, which in turn play a significant role in biological functions. Especially the formation of apoM-bound S1P is of physiological significance. Striking progress has been made in the last decade indicating that S1P is an important mediator in the liver, regulating a variety of hepatic functions. The current understanding of S1P signaling in the liver indicates that S1P affects several cell types of the liver and that a dysregulation of S1P metabolism and signaling occurs under pathophysiological conditions. However, the role of S1P on hepatic function is not completely elucidated, as both protective and harmful functions of the sphingolipid have been reported. In particular, the function of S1P seems to be controversial depending on the liver cell type and the S1P receptor expression profile. Further studies are needed to delineate the critical role of S1P in liver physiology and pathophysiology such as insulin resistance, NAFLD, and HCC. After careful consideration of the connection between S1P and liver pathophysiology, it seems likely that S1P is an accomplice in several liver diseases. All in all, it can be speculated that S1P signaling is a promising target for the development of novel therapies for the treatment of a variety of liver diseases. The development of targeted pharmacological interventions modulating S1P signaling is being actively investigated, and it can be hoped that this will provide useful therapeutics.

## Abbreviations

S1P	Sphingosine 1-phosphate
SphK	Sphingosine kinase
SPNS2	Protein spinster homologue 2
ABC	ATP-binding cassette
S1PR	Sphingosine 1-phosphate receptor
TNF	Tumour necrosis factor
HDAC	Histone deacetylase
apoM	Apolipoprotein M
HFD	High-fat diet
GSK	Glycogen synthase kinase
NAFLD	Non-alcoholic fatty liver disease
NASH	Non-alcoholic steatohepatitis
TLR	Toll-like receptor
PKC	Protein kinases C
PPARγ	Proliferator-activated receptor γ
α-SMA	Alpha-smooth muscle actin
HSCs	Hepatic stellate cells
HCC	Hepatocellular carcinoma
TGF	Transforming growth factor
DENV	Dengue virus
HBV	Hepatitis B virus
HCV	Hepatitic C virus
BVDV	Bovine viral diarrhea virus
HBx	HBV X protein
DDR	DNA damage response
